# Recent Advances in Diamond-Capped GaN HEMTs for RF Application

**DOI:** 10.3390/nano16040224

**Published:** 2026-02-09

**Authors:** Yuanmeng Xiang, Mei Wu, Haolun Sun, Shiming Li, Hongda Chen, Jiamin Wei, Binyan Yan, Ling Yang, Meng Zhang, Hao Lu, Bin Hou, Xiaohua Ma, Yue Hao

**Affiliations:** National Engineering Research Center of Wide Band-Gap Semiconductor, Faculty of Integrated Circuit, Xidian University, Xi’an 710126, China

**Keywords:** GaN HEMTs, TBR, diamond film, thermal management

## Abstract

Self-heating effects severely limit the performance of gallium nitride high-electron-mobility transistors (GaN HEMTs) in high-power radio frequency (RF) applications. Diamond capping technology leveraging diamond’s exceptional thermal conductivity (>2000 W/m·K) has emerged as a highly promising near-junction cooling solution. However, its integration with GaN HEMTs faces challenges including lattice/thermal mismatch, high thermal boundary resistance (TBR), and process compatibility. This review summarizes recent progress in high-thermal-conductivity diamond film growth, TBR optimization, thermal simulations, and the integrated process with GaN devices. These technological breakthroughs enable diamond-capped GaN HEMTs with an excellent comprehensive performance. Continued advances in these fields will be critical for fully releasing the capabilities of diamond capping technology for GaN HEMTs in high-frequency and high-power applications.

## 1. Introduction

Gallium nitride (GaN), a leading representative of third-generation semiconductor materials, exhibits tremendous application potential in high-power and high-frequency electronic devices due to its excellent properties, such as its wide bandgap, high critical breakdown electric field, and high electron mobility [1,2,3,4]. GaN high-electron-mobility transistors (HEMTs) have become core components in modern electronic systems, such as 5G communication base stations, high-power fast charging systems, and satellite communication systems [5,6,7].

In 1994, Khan et al. first reported the frequency characteristics of GaN HEMTs with a gate length of 0.25 μm, achieving a cutoff frequency (f_T_) of up to 11 GHz and a maximum oscillation frequency (f_max_) of 35 GHz [8]. In 2004, Y.-F. Wu et al. demonstrated a power density of 32.2 W/mm and a power-added efficiency (PAE) of 54.8% at 4 GHz [9]. With the continuous advancement of technology, significant progress has been made in the power performance research of GaN HEMTs to date. Marko J. Tadjer et al. achieved a direct current (DC) output power density (P_OUT_) of 56 W/mm at 4 GHz (C-band) [10]. Hong Zhou et al. from Xidian University realized output power densities of 42 W/mm and 20 W/mm at 8 GHz and 30 GHz, respectively, which represent a 30% and 43% improvement over the previous highest power densities reported for all X-band and Ka-band transistors [11]. However, with the continuous increase in device power density, the heat generated internally has accumulated dramatically, leading to severe self-heating effects [12,13]. The self-heating effect significantly increases the junction temperature of the channel in GaN HEMTs, often exceeding the optimal operating range of gallium nitride materials and thereby restricting the performance of GaN HEMTs. First, high temperatures intensify phonon scattering, reducing the electron mobility in the two-dimensional electron gas (2DEG) channel and subsequently leading to the degradation of drain current and transconductance (g_m_) [14]. Meanwhile, the thermal activation of deep-level traps and temperature-dependent changes in polarization effects induce threshold voltage drift, undermining the stability of the device’s switching characteristics [15]. Finally, long-term operation in a high-temperature environment accelerates the degradation of GaN HEMTs, including the increase in interface defects and the failure of passivation layers, which severely impairs device reliability and shortens its operational lifetime [16]. Consequently, efficient thermal management has become a critical challenge to unlock the full potential of GaN devices and ensure their long-term reliable operations.

To address the self-heating issue in GaN HEMTs, researchers are actively developing efficient thermal management solutions. Among thermal management materials, diamond has attracted significant interest due to its exceptional thermal properties. It possesses the highest thermal conductivity (TC), ~2000 W/m·K, along with an excellent chemical stability and electrical insulation, making it the ultimate heat dissipation material [17,18]. Two main technical routes have been explored for integrating diamond with GaN devices. One is using diamond as a substrate for fabricating GaN-on-diamond structures, while the other involves depositing diamond film as a capping layer on GaN HEMTs [19,20,21]. As a near-junction cooling strategy, this configuration offers several key advantages, including a shorter heat dissipation path with a higher cooling efficiency [22,23].

Despite these advantages, the practical implementation of diamond-capped technology still faces three major challenges. The first is the significant lattice mismatch and thermal mismatch between diamond and GaN, which lead to the deterioration of interface bonding quality [24,25]. Introducing interlayers is an effective solution to protect the heterojunction. Common interlayer materials include SiN_x_, SiO_2_, and Al_2_O_3_, which can improve the bonding compatibility but introduce a high thermal boundary resistance (TBR) [26,27,28]. Second, the actual TC of polycrystalline diamond (PCD) films grown via chemical vapor deposition such as microwave plasma chemical vapor deposition (MPCVD) is considerably lower than that of bulk single crystal diamond. Influenced by factors such as grain size and impurity, it typically ranges from several hundred to 1000 W/(m·K), thereby constraining the overall cooling efficiency [29,30,31,32]. Finally, there is the compatibility issue between the diamond growth process and the GaN HEMT fabrication process. Diamond deposition usually requires high temperatures at more than 700 °C and a hydrogen-rich plasma environment, which can etch the GaN surface, degrade 2DEG properties, and damage pre-existing metal gates and ohmic contacts [33,34,35]. These interrelated issues collectively hinder the performance improvement and long-term reliability of diamond-capped GaN HEMTs.

This paper presents a systematic review of recent progress in diamond-capped GaN HEMT technology. The discussion is organized into three parts. First, it explores diamond growth optimizations and TBR reduction strategies, including improvements in MPCVD systems, innovations in nucleation techniques, the precise control of growth parameters such as temperature, pressure, and power, interlayer material optimization, and interface nanostructuring. Second, it summarizes key findings from device simulation studies that reveal the influence of critical parameters (TC and TBR) on electro-thermal performance and analyzes the inherent trade-off between thermal optimization and high frequency characteristics. Third, it compiles recent experimental results from the two primary integration routes, the gate-first and gate-last approach, and compares their performance improvements in terms of DC characteristics, radio frequency (RF) performance, power handling capability, and operational reliability. By synthesizing important research findings from recent years, this paper aims to provide a clear framework for understanding the current status, existing challenges, and future development directions of diamond-capped technology, offering valuable insights for the development of next-generation high-performance, high-reliability GaN electronic devices.

## 2. Diamond Film Growth and Interface Optimization

When directly growing a diamond thin film on the top side of GaN HEMTs, the TC of the diamond layer is the primary consideration. Studies have shown that the TC of diamond films fabricated via MPCVD is significantly influenced by factors such as grain size and impurity. Therefore, achieving a high TC for thin films is critical for the growth of high-performance diamond films. Additionally, the TBR at the diamond/GaN interface represents another key factor limiting heat dissipation performance. A high TBR can severely hinder interfacial heat transfer, which accumulates heat in the GaN channel and aggravates self-heating in GaN HEMTs. This is mainly attributed to the large lattice mismatch between GaN and diamond, which generates a high density of defects and voids at the interface and further increases thermal resistance [36]. Furthermore, the significant difference in thermal expansion coefficients between GaN (5.2 × 10^−6^ K^−1^) and diamond (0.7 × 10^−6^ K^−1^) introduces substantial residual stress in the GaN layer during growth or subsequent thermal cycling, which can degrade material quality and exacerbate interfacial imperfections [37,38,39]. The TBR is also associated with the acoustic mismatch between GaN and diamond, as well as the formation of defective transition regions near the nucleation surface [40]. To protect the GaN HEMTs from hydrogen plasma etching during diamond growth, an interlayer is often deposited on the GaN surface prior to diamond growth. However, this interlayer introduces additional TBR, which severely compromises the device’s cooling efficiency.

Current research mainly focuses on two aspects: on the one hand, improving the quality and TC of the diamond film by optimizing the MPCVD process parameters such as temperature, chamber pressure, plasma power, and gas atmosphere; on the other hand, reducing TBR by thinning the interlayer, optimizing the growth conditions of the interlayer material, or designing novel interface structures.

### 2.1. Optimization of Diamond Film Growth

#### 2.1.1. Nucleation Technology

MPCVD technology has become an important method for preparing high-quality diamonds due to its precise growth process control, clean deposition environment with extremely low pollution risk, and large-area deposition capacity [41]. In this process, nucleation is the first crucial step for the growth of diamond thin films. Due to the difficulty of spontaneous diamond nucleation, auxiliary nucleation techniques must be employed to improve nucleation quality and efficiency. The evolution of nucleation technology has progressed from the early grinding method to the colloid chemistry-optimized electrostatic seeding method. A range of pretreatment strategies for boosting diamond nucleation on target substrates are commonly employed in related research. Grinding is a basic approach that introduces surface defects or embeds diamond particles by physically scratching the substrate [42,43]. Electrostatic seeding relies on colloid chemistry principles to modulate the zeta potential of nanodiamond particles so that a uniform adsorption of the particles on the substrate can be achieved [44]. Chemical nucleation is a specialized technique that adopts small molecules like diamondoids as nucleation embryos and forms uniform nucleation sites at low temperatures through chemical bonding or deposition processes [45]. Bias-Enhanced Nucleation (BEN) involves applying a negative DC bias of 100 to 250 V to the substrate in a CVD chamber filled with a CH_4_-rich atmosphere, and the applied electric field generated in this way can effectively promote the adsorption and nucleation of gas-phase precursors such as CH_3_· radicals [46]. Yongbing Tang et al. analyzed the advantages and disadvantages of the aforementioned methods and finally drew the conclusion that electrostatic seeding is significantly superior to other approaches in terms of nucleation density and uniformity, serving as the preferred strategy for the preparation of ultrathin diamond films [47]. The remaining techniques possess unique merits in specific application scenarios, yet all of them have inherent limitations regarding substrate compatibility or process complexity.

**Figure 1:**
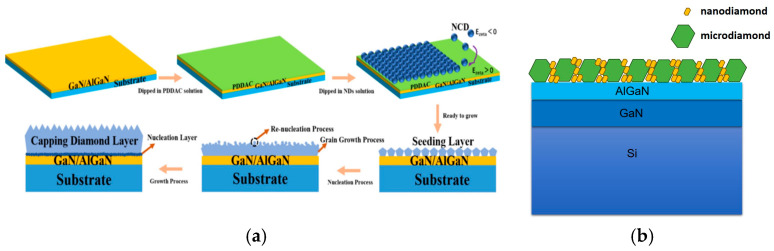
(**a**) Polymer-assisted dip seeding and diamond growth principle (Copyright 2021, American Chemical Society, reprinted from [49] with permission). (**b**) Microdiamond–nanodiamond mixed-seeding on GaN/Si substrate.

Currently, electrostatic seeding is the most widely used method for enhancing the nucleation of diamond thin films [48]. As an effective nucleation approach for growing diamond films on AlGaN/GaN structures, it enables the formation of a more uniform seeding layer, thereby reducing residual stress during diamond deposition. For example, Xiaobo Hu et al. from Shandong University adopted electrostatic adsorption seeding [49]. Figure 1a schematically illustrates the polymer-assisted dip seeding and capping process of diamond growth, clearly showing the electrostatic adsorption of nanodiamond particles on the GaN substrate to form a uniform seeding layer. The technique aims to reduce residual stress and minimize non-diamond phases. The diamond film deposited via electrostatic seeding exhibits a dense and uniform morphology without obvious agglomeration. Residual stress is concentrated in the interfacial nucleation region, and the compressive stress of diamond is reduced to 0.09 GPa. Additionally, the weak G band (~1800 cm^−1^) in the Raman spectrum indicates the suppression of sp^2^-bonded non-diamond phases [50].

In addition to conventional auxiliary nucleation methods, hybrid seeding technology, with the combination of microscale and nanoscale seed crystals, has been employed. This approach can not only protect the GaN layer but also reduce the overall TBR of GaN HEMTs, thereby enabling the efficient heterogeneous integration of diamond and GaN. In 2020, P.W. May et al. developed a diamond nucleation scheme based on mixed-seeding under a ~35 kV electrostatic spray [51]. As illustrated in Figure 1b, this scheme involves a two-step process. First, microdiamond seed crystals with a size of 2.0 ± 1.0 μm are deposited on the substrate surface via electrostatic spray technology; 10 mg of microdiamond powder is adopted, and the spray operation is carried out two times to establish a low-thermal-resistance interface. Subsequently, secondary seeding is performed using nanodiamond crystals with a size of 3.3 ± 0.6 nm; 50 drops of 1 wt% nanodiamond aqueous dispersion are used, and the spray operation is implemented two times to fill the gaps between the microdiamond particles. Key parameters such as the concentration ratio of microdiamond and nanodiamond seed crystals and the number of spray cycles are further optimized, ultimately achieving dense single-layer seed crystal coverage. The results demonstrate that the incorporation of nanodiamond seed crystals can effectively eliminate interfacial voids, prevent the substrate from being etched during subsequent processes, and thus form a complete interfacial nucleation structure.

#### 2.1.2. Growth Stage Optimization

**Figure 2:**
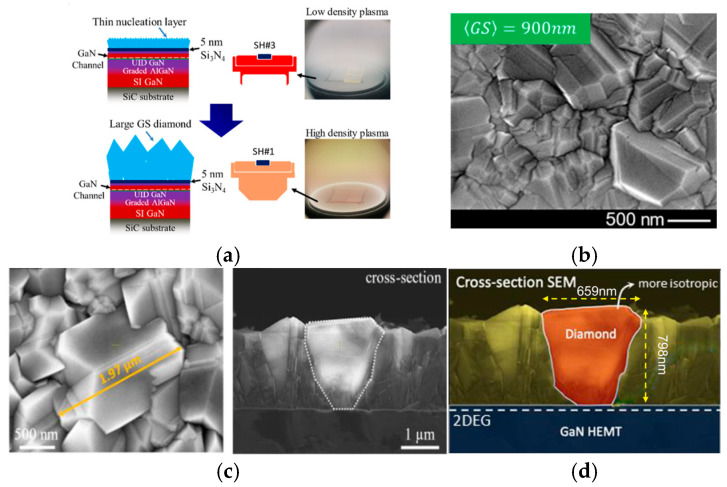
(**a**) Schematic of the two-stage process for diamond growth (from M. Malakoutian, 2021 doi:10.1021/acs.cgd.0c01319, CC-BY-NC-ND, reprinted from [32] with permission). (**b**) SEM image of the diamond film prepared using the two-stage process; average grain size 900 nm [32]. (**c**) High-density plasma growth showing 2 μm diamond with more isotropic grains (from M. Malakoutian, 2021, doi:10.1021/acsami.1c13833, CC-BY-NC-ND 4.0, reprinted from [52] with permission). (**d**) SEM micrographs of diamonds grown at 400 °C [52].

Diamond growth parameters play a crucial role in enhancing the TC of diamond films, primarily including growth temperature, microwave plasma power, chamber pressure, and gas atmosphere. Srabanti Chowdhury et al. from Stanford University innovatively proposed a two-stage growth process under high temperature [32]. The core design of this process is to decouple the nucleation stage from the main growth stage for targeted diamond growth. As illustrated in Figure 2a, during the nucleation stage, a low power of 600 W and a low pressure of 20 Torr are adopted, with a growth temperature of 700 °C. This mild plasma environment ensures the rapid formation of a dense thin diamond nucleation layer to shield the underlying GaN surface from subsequent high-energy plasma etching. During the main growth stage, the plasma power and chamber pressure are ramped up to 1600 W and 70 Torr, respectively. The high-density hydrogen plasma under these parameters can enhance the formation of sp^3^-bonded carbon and promote the selective etching of sp^2^-bonded carbon, thereby significantly improving the diamond phase purity and TC. The anisotropy ratio was defined here as a key parameter reflecting the quality of diamond film, which refers to the ratio of diamond film thickness to its average grain size. The final fabricated diamond film, as shown in Figure 2b, exhibits an average grain size of 900 nm, and its anisotropy ratio is reduced to 1.12–1.7. This structural optimization directly boosts the TC of the diamond film, reaching ~400 W/(m·K) for a 600 nm diamond and ~600 W/(m·K) for a 900 nm diamond. Ultimately, when this high-quality diamond film is integrated onto GaN HEMTs, the channel temperature can be reduced by up to 150 °C at a power density of 15 W/mm, effectively mitigating the self-heating issue of GaN HEMTs and improving their high-frequency and high-power operation stability.

In addition, Dong et al. analyzed the correlation between the TC and grain size of diamond films [53]. They pointed out that the TC of diamond films increases with the increase in grain size. When the grain size of diamond is in the range of 1–2 μm, the TC of the film can reach 500–600 W/(m·K). Subsequently, Srabanti Chowdhury et al. further optimized the diamond nucleation process to achieve high-quality PCD [52]. The key to achieving this high TC lies in the use of a relatively higher power and pressure (1800 W and 70 Torr) during the main growth stage. As shown in Figure 2c, the grown grains tend to be isotropic, with similar transverse and longitudinal dimensions up to approximately 2 μm. Large transverse grains can reduce the phonon scattering and improve in-plane TC, and this higher in-plane TC further enhances the overall TC of the diamond film. As a result, The TC of the 2 μm thick diamond film reached 638 ± 48 W/(m·K). Moreover, this research team proposed a three-step low-temperature diamond growth process in 2022 [35]. The innovation of this process lies in employing customized CH_4_/O_2_ ratios at different growth stages. The core of the process is the introduction of O_2_, which creates OH radicals in the plasma [54,55]. Compared to hydrogen atoms, OH radicals have a stronger etching effect and display a more distinct selective etching ability toward sp^2^-bonded carbon [56]. At a low temperature of 400 °C, this process successfully produces high-quality diamond. As illustrated in Figure 2d, the diamond film features an average grain size of 659 nm with a maximum phase purity of 97.1% and a low anisotropy ratio of 1.21. It brings a relatively high TC of 300 W/(m·K) under this thickness. The Raman spectra of diamonds grown under different methane (CH_4_) concentrations were conducted. During the first-step nucleation process, a low CH_4_ volume proportion of 3.4% is helpful to realize uniform thin film growth, and it leads to the formation of a narrower sp^3^ peak with a smaller full width at half-maximum (FWHM) of only 6.6 cm^−1^. This method successfully enables the growth of high-quality diamond films at low temperatures, which is more conducive to the compatible integration of diamond films with GaN HEMTs.

**Figure 3:**
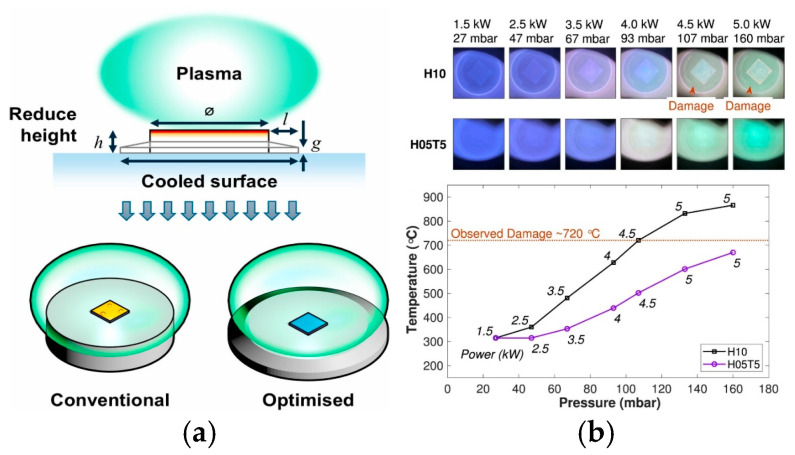
MPCVD-related designs and damage characterizations for thick diamond deposition on flipped III-N/GaN on Si (from Cuenca, J.A., 2025, doi:10.1016/j.carbon.2025.120349, CC-BY, reprinted from [58] with permission): (**a**) schematics of conventional cylindrical holder and optimized tapered holder; (**b**) sample photographs at various microwave power densities (MWPD) and the influence of power on temperature before and after optimization.

Optimizing MPCVD equipment is also an effective strategy for improving diamond growth quality [57]. In 2025, Oliver A. Williams et al. from Cardiff University designed a novel conical scaffold named H05T5 using microwave plasma modeling methods [58]. The design principle is based on the optimization of thermal equilibrium equations, where the substrate temperature is determined by the dynamic balance between plasma heating and cooling dissipation. As illustrated in Figure 3a, the height of the designed conical scaffold is half that of the conventional ones, which promotes a more uniform plasma distribution and mitigates edge hotspot formation. Concurrently, the conical structure increases the cooling surface area, facilitating a faster heat diffusion to the environment and enhancing cooling flux. As shown in Figure 3b, under high-power plasma conditions of 5 kW/160 mbar, the substrate temperature was effectively controlled below 669 °C, with GaN damage in the plasma environment successfully avoided. Beyond avoiding substrate damage, this scaffold also optimizes key growth metrics and elevates film quality. Its average growth rate reaches 1.95 μm/h at the second stage, nearly doubling that of the traditional H10 holder with 1.05 μm/h. Moreover, optical microscopy results confirm that the deposited films have grain sizes larger than 20 μm.

### 2.2. Optimization of TBR at the Diamond/GaN Interface

The approaches to optimize TBR mainly fall into two categories: one is to reduce the thermal resistance of the interlayer itself, such as by using materials with higher TC or thinning the thickness of the interlayer. Another approach is to optimize the interface structures, such as by altering the interface structure to increase the contact area, thereby reducing phonon scattering and lowering the contact thermal resistance.

#### 2.2.1. Interlayer Optimization

**Figure 4:**
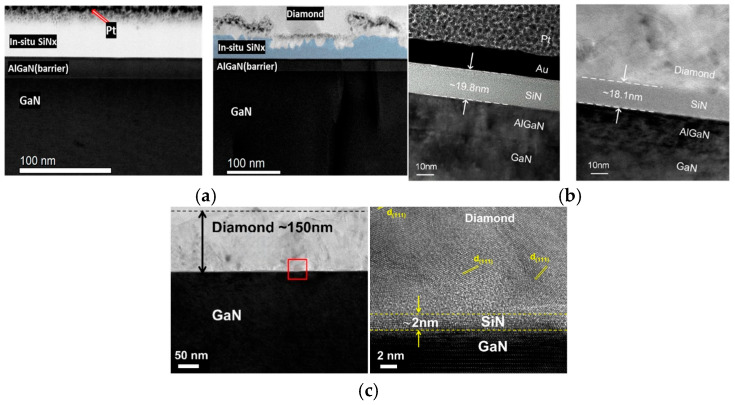
(**a**) Scanning Transmission Electron Microscope (STEM) images showing the AlGaN/SiN_x_ layers before (**left**) and after (**right**) diamond growth (Copyright 2019, American Chemical Society, reprinted from [48] with permission). (**b**) TEM images of the interface before and after diamond growth (Copyright 2022, AIP Publishing, reprinted from [60] with permission). (**c**) TEM image of the GaN/SiN_x_ layer after diamond growth (Copyright 2024, Elsevier, reprinted from [62] with permission). The red box in the left panel indicates the magnified region shown in the right panel.

Optimizing the material type and thickness of the interlayer can significantly reduce the TBR and enhance the heat dissipation performance of GaN HEMTs. Researchers have found that, although AlN exhibits a higher TC than SiN_x_, it fails to form a uniform and continuous coating when used as an interlayer, resulting in a significantly higher TBR compared to the SiN_x_ interlayer [59]. For the SiN_x_ interlayer, in 2019, Edwin L. Piner et al. from Texas State University prepared an in situ SiN_x_ interlayer via metal–organic chemical vapor deposition (MOCVD) [48]. As shown in Figure 4a, they achieved a TBR of 52.8 m^2^·K/GW with a 20 nm SiN_x_ interlayer. In 2022, Mei Wu et al. from Xidian University integrated a 1.5 μm thick PCD thin film as a heat spreader and a 20 nm plasma-enhanced chemical vapor deposition (PECVD) SiN_x_ as an interlayer on the AlGaN/GaN heterojunction on SiC to fully leverage the ultrahigh TC of diamond for device-level cooling [60]. The PECVD SiN_x_ interlayer serves dual functions as a nucleation layer for diamond growth and a passivation layer for the AlGaN/GaN heterojunction. The diamond exhibits a high purity, with a strong characteristic peak at 1333.6 cm^−1^, while the non-diamond peak formed by graphite between 1500 and 1600 cm^−1^ has a negligible intensity. The E_2_ (high) peak of GaN shows a redshift of 0.42 cm^−1^ corresponding to an increase in tensile strain. Diamond deposition increases the tensile strain of the GaN layer from 58.14 MPa to 155.81 MPa, thereby increasing the 2DEG density by 4.9% from 1.03 × 10^13^ cm^−2^ to 1.08 × 10^13^ cm^−2^ without compromising its intrinsic performance. Meanwhile, as shown in Figure 4b, the thickness of the PECVD SiN_x_ layer decreased from 19.8 nm to 18.1 nm after diamond growth, and energy dispersive X-ray spectroscopy (EDS) reveals that a transition layer forms at the diamond/SiN_x_ interface. Electron energy loss spectroscopy (EELS) analysis confirms that this transition layer is a thin SiC layer formed through the replacement of N atoms in SiN_x_ with C atoms. In addition, the effective TC of diamond measured through time domain thermal reflectance (TDTR) is 313.68 W/m·K, and the TBR between diamond and GaN is 39.35 m^2^·K/GW. The thermal simulation shows a 17.1 °C temperature reduction of the device at a power density of 15 W/mm, with diamond only covering the access region. As shown in Figure 4c, Junjun Wei et al. from the University of Science and Technology Beijing achieved a TBR of 12.8 ± 0.64 m^2^·K/GW by thinning the SiN_x_ protective layer from the initial 10 nm to about 2 nm [60]. The average grain size of diamond film is 20 nm and the film thickness is 150 nm. Meanwhile, the TC of diamond film has reached 200 ± 40 W/(m·K). In addition, SiN_x_ can effectively reduce residual stress and deformation in diamond films. Chengming Li et al. investigated the stress-relief effect of an SiN_x_ interlayer [61]. The experimental characterization results demonstrate that the SiN_x_ interlayer can reduce the warpage of the grown diamond film. The warpage in the direction perpendicular to the film is reduced by 22.63%, and the overall stress calculated via the Stoney formula drops from 0.42 GPa to 0.33 GPa, leading to a reduction of 21.43%.

**Figure 5:**
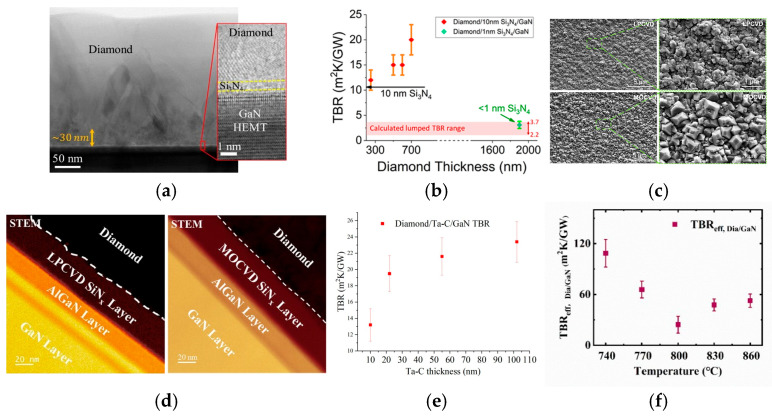
Sample characterization and TBR properties of diamond/SiN_x_/GaN structure [52]: (**a**) STEM analyses showing a 30 nm nucleation layer and <1 nm SiN_x_; (**b**) TBR with two SiN_x_ thicknesses vs. diamond layer thickness. Growth conditions of SiN_x_ interlayers and STEM analyses of samples with rough interfaces (Copyright 2022, Wiley, reprinted from [64] with permission): (**c**) under LPCVD and MOCVD growth conditions; (**d**) STEM analyses of sample L and sample M, both with rough interfaces. (**e**) TBR of diamond/ta-C/GaN at different ta-C film thicknesses (Copyright 2025, Elsevier, reprinted from [66] with permission). (**f**) TBR_eff, Dia/GaN_ for samples with different growth temperatures (Copyright 2025, Elsevier, reprinted from [67] with permission).

In 2021, Srabanti Chowdhury et al. developed an innovative diamond integration scheme [52]. By adopting a low-density plasma condition (600 W, 20 Torr) for nucleation, the etching rate of the SiN_x_ interlayer by plasma was precisely controlled. The initial SiN_x_ thickness is 5 nm; due to the growth control, an ultrathin and intact SiN_x_ interlayer of less than 1 nm remained after the diamond growth, as shown in Figure 5a. In addition, the diffusion of C forms SiC at the interface, creating a compositionally graded transition zone within SiN_x_. These factors together yield a record-low TBR of 3.1 ± 0.7 m^2^·K/GW, as illustrated in Figure 5b. As noted previously, carbon diffusion transforms the surface SiN_x_ layer into a thin SiC layer, creating a compositionally graded transition region that minimizes phonon reflection effectively. This distinctive structural design is critical, as it contributes to the low TBR when SiN_x_ acts as an interlayer [59,63]. In 2023, Haolun Sun et al. from Xidian University compared SiN_x_ interlayers with thicknesses of 100 nm, 40 nm, and 40 nm via PECVD, low-pressure chemical vapor deposition (LPCVD), and metal–organic chemical vapor deposition (MOCVD), respectively [64]. Regardless of whether the SiN_x_ was deposited at 250 °C or 350 °C, the interlayer melted on the GaN surface after diamond growth, leading to a discontinuous and uneven PCD film coverage. Figure 5c displays that the sample with SiN_x_ grown using LPCVD and MOCVD achieved a uniform and full diamond film coverage on the SiN_x_ surface without any visible damage to the interlayer across different observation scales. SiN_x_ grown using PECVD could not withstand the diamond growth temperature and melted, with a measured TBR of approximately 40 m^2^·K/GW. The growth temperature of MOCVD reaches 900 °C, which is significantly higher than that of LPCVD with 700 °C and PECVD with 250–350 °C. A high temperature provides a sufficient thermodynamic driving force for the migration of C atoms. Meanwhile, the STEM images in Figure 5d confirm that the interface of MOCVD and diamond is smoother, while it shows a relatively rough interface for the LPCVD sample. The lowest TBR of ~24.6 m^2^·K/GW was achieved among the three samples using the MOCVD SiN_x_ interlayer. These comparative experiments demonstrate that MOCVD SiN_x_ exhibits a superior performance in reducing the TBR of diamond/GaN heterostructures. While C diffusion in the SiN_x_ interlayer contributes to TBR reduction, there is a limit to such diffusion. The pursuit of an even lower TBR has motivated researchers to explore novel materials with a higher intrinsic TC and more stable interfaces. In 2023, Srabanti Chowdhury et al. employed molecular dynamics simulations and further proved that an amorphous SiC interlayer is helpful for the reduction of TBR [65]. This is mainly because SiC has a highly matched phonon density of states with diamond.

Moreover, Kesheng Guo et al. selected tetrahedral amorphous carbon (ta-C) as the interlayer between diamond and GaN, achieving a low TBR of 13 m^2^·K/GW for diamond/ta-C/GaN when the thickness of the ta-C interlayer is 10 nm [66]. As shown in Figure 5e, the TBR of diamond/ta-C/GaN showed a significant decrease when the thickness was less than 20 nm. They found that, during the MPCVD process, hydrogen plasma can convert 2–3 nm of ta-C into ultra-nanocrystalline diamond (UNCD). The UNCD fuses with the nucleated and grown diamond, which effectively mitigates the thermal expansion coefficient mismatch and reduces the thermal resistance at the interface. In addition to the influence of the interlayer on TBR mentioned above, Chao Yuan et al. from Wuhan University found in 2024 that the diamond growth temperature also affects TBR [67]. They systematically investigated the effect of growth temperature in the range of 740 °C to 860 °C on the thermal properties of the diamond/SiN_x_/GaN. The minimum effective TBR for the diamond/GaN (TBR_eff, Dia/GaN_) of ~24 m^2^·K/GW was achieved at 800 °C, while the highest TBR_eff, Dia/GaN_ of ~96 m^2^·K/GW was achieved at 740 °C. These TBR_eff, Dia/GaN_ variations with growth temperature are clearly visualized in Figure 5f, which quantifies the trend of TBR_eff, Dia/GaN_ first decreasing and then increasing as temperature rises from 740 °C to 860 °C. At 740 °C, insufficient amorphous carbon etching and carbon diffusion induce a 120 ± 5 nm transition layer with a complex microstructure including amorphous SiN_x_, grain boundaries, interfacial defects, and SiC. At 860 °C, GaN lattice distortion and interfacial graphitization elevate the TBR_eff, Dia/GaN_ to ~60 m^2^·K/GW, significantly higher than the minimum TBR_eff, Dia/GaN_ achieved at 800 °C. This is attributed to the presence of graphite phases in the transition layer and the deviation of the lattice spacing of the GaN (100) crystal plane from its standard value of 2.76 Å to 3.6 Å [68]. Device simulations based on the optimized growth parameters at 800 °C demonstrate that a 1.5 μm thick diamond layer can achieve a maximum 23% reduction in the device’s peak temperature. Meanwhile, the transition layer thickness at this optimal temperature is only 35 ± 5 nm, much thinner than the 120 ± 5 nm at 740 °C and 80 ± 10 nm at 860 °C, and the diamond heat dissipation layer can reduce the maximum active region temperature from 185 °C to 142.3 °C, validating its thermal mitigation effect.

#### 2.2.2. Interface Structure Optimization

**Figure 6:**
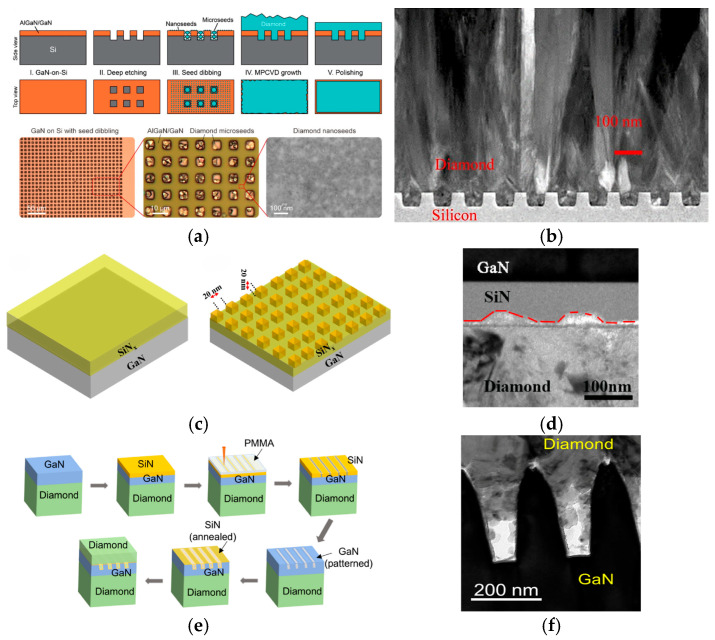
(**a**) Schematic diagram of the mixed seed crystal growth method (Copyright 2021, ACS, reprinted from [71] with permission). (**b**) TEM image of the patterned diamond–silicon interface (Copyright 2019, ACS, reprinted from [72] with permission). (**c**) Schematic of GaN/SiN_x_ with (**right**) and without (**left**) periodic array structure (from Jia, X., 2021, doi:10.3390/coatings12050672, CC-BY 4.0, reprinted from [73] with permission). (**d**) TEM micrographs of GaN/SiN_x_/diamond interface [73]. Patterned process flow and structural characterization (from Ji, X., 2025, doi:10.1021/acsaelm.5c00119, CC-BY 4.0, reprinted from [74] with permission): (**e**) schematic of the patterned process flow; (**f**) TEM image of the patterned structure.

In addition to optimizing the interlayer material, increasing the interfacial contact area by introducing an interfacial microstructure can enhance phonon transmission probability, strengthen interfacial adhesion, reduce residual stress, alleviate interfacial stress concentration and simultaneously decrease the TBR [69,70]. Reza Soleimanzadeh and colleagues from the École Polytechnique Fédérale de Lausanne (EPFL) in Switzerland adopted deep etching to create micrometer-scale hole arrays on the substrate [71]. Each hole featured a width of approximately 4 μm and a depth of around 5 μm. Subsequently, they employed mixed diamond seeding using micro-seeds and nano-seeds. As illustrated in Figure 6a, this structural design allows for the effective confinement of microscale seeds inside the holes. In the meantime, nanoscale seeds ensure a full area coverage across the entire substrate surface. The diamond grown from nano-seeds filled up the spaces between the micro-seeds and the side walls, and the high seeding density enabled the rapid initial growth of a protective diamond film, which further prevented the GaN substrate from being eroded by hydrogen plasma during the subsequent processes. Notably, this fabrication route yielded a smooth interface between diamond and GaN with no observable damage to the GaN or its underlying buffer layer. Moreover, the diamond pillars embedded in the substrate markedly boosted the adhesion of the diamond film to the underlying substrate. Raman spectroscopy measurements confirmed that the residual stress of the diamond film prepared using this scheme was as low as 0.2 GPa, a value far below the typical residual stress of more than 1.5 GPa for diamond films fabricated through traditional seeding techniques.

Furthermore, researchers extended the concept of arrayed microstructure regulation to the field of thermal transport performance optimization for diamond heterointerfaces. Samuel Graham et al. from the Georgia Institute of Technology proposed a method to regulate the diamond/Si interface using nanopatterned array structures for enhancing interfacial thermal conductance (TBC), with the structure shown in Figure 6b [72]. The array is nanoscale trenches with trapezoidal cross-sections. Sample A2 has a trench height of 47 nm, top width of 60 nm and bottom width of 77 nm, while sample B2 has a trench height of 105 nm, top width of 205 nm and bottom width of 215 nm. The test results demonstrate that the magnitude of TBC improvement is highly consistent with the extent of the contact area expansion. The TBC without an array structure is 63.7 MW/m^2^·K. For sample A2, the TBC increases to 105 MW/m^2^·K, and its diamond cross-plane TC is 28% higher than that of the flat sample. Meanwhile, the TBC of sample B2 rises to about 80.3 MW/m^2^·K, with its diamond cross-plane TC 10% higher than that of the flat sample.

In 2022, Lu Huang et al. also found that periodic array structures can effectively reduce the TBR between diamond and GaN, and this reduction is attributed to the increased contact area, enhanced interfacial bonding strength, and improved diamond nucleation density [73]. The morphology of the periodic array structures is 20 nm × 20 nm cubic pits on the 100 nm thickness SiN_x_ interlayer, as shown in Figure 6c,d. The critical load (Lc_3_) of the periodic array structure sample increased from 8 N to 15 N, demonstrating an enhanced interfacial bonding strength. This can be directly verified using transmission electron microscopy (TEM) observations. The interface of the periodic structure forms a wave-like mosaic structure, where diamond particles interlock with the SiN_x_ layer. This not only hinders the propagation of transverse cracks in the film but also reduces interfacial voids through mechanical interlocking. Diamond nucleation density analysis shows that diamond particles on the surface of periodic samples are denser. Consequently, TBR decreased from 40.5 ± 2.5 m^2^·K/GW of the non-periodic array structure to 32.2 ± 1.8 m^2^·K/GW of the periodic array structure. In 2025, Martin Kuball et al. further proposed a method to increase the contact area between GaN and diamond, reducing the TBR of diamond/GaN from 51 m^2^·K/GW to 25 m^2^·K/GW [74]. The schematic diagram of its process flow is shown in Figure 6e; they first etched grooved structures with controlled spacing on GaN, followed by the rapid thermal annealing of a 10 nm PECVD SiN_x_ interlayer at 1000 °C. A diamond film with a thickness of 1 μm was subsequently deposited via MPCVD. An effective control of the TBR was achieved by adjusting the grooved structure and groove spacing to modify the contact area between GaN and diamond. As presented in Figure 6f, the groove pitch is 200 nm and trench depth is 320 nm, and diamond only fills 20–80% of the trench volume. This incomplete filling stems from the coalescence of diamond seeds at the trench top limiting bottom growth. When the grooves are fully filled, the actual contact area between GaN and diamond will be larger, leading to an even more significant TBR reduction.

## 3. Simulation of Diamond-Capped GaN HEMTs

Device simulation plays an indispensable role in the research of diamond-capped GaN HEMTs. It not only serves as a link between material properties and device performance, but also enables a low-cost assessment of the impact of different structural designs, material parameters and interface characteristics on the electrothermal performance of devices before expensive device fabrication, thereby guiding experiments and reducing R&D costs. The core purpose of simulation is to explain how to convert the thermal and electrical parameters of materials into improvements in device performance and reliability. This section will elaborate on the influence of material parameters such as the TC, thickness, dielectric constant, and TBR on the thermal characteristics, DC characteristics, and RF characteristics of devices.

**Figure 7:**
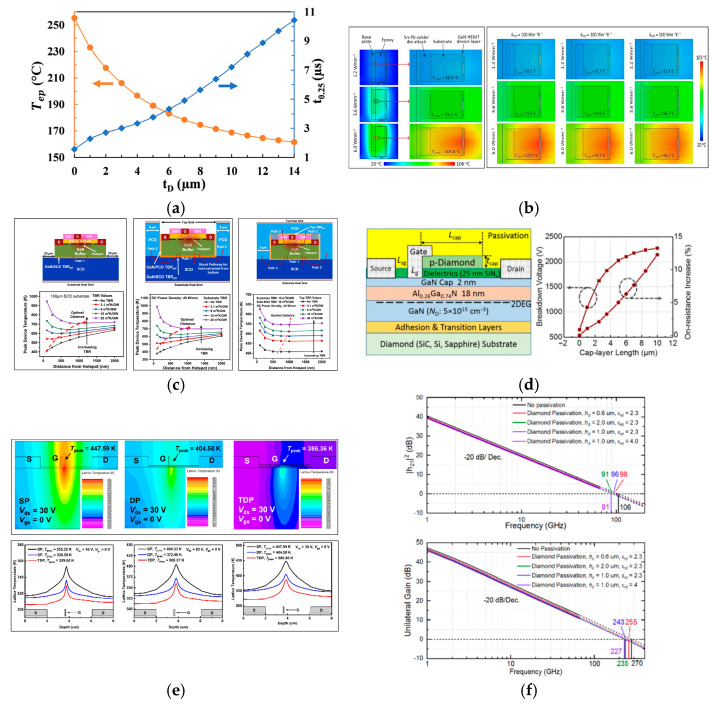
(**a**) Effect of diamond film thickness on T_ep_ and t_0.25_ (Copyright 2021, IEEE, reprinted from [82] with permission). (**b**) Temperature distribution inside the original TO-220 packaged Si-GaN-SiN_x_ chip (**left**) and Si-GaN-PCD chip (**right**) (Copyright 2024, Elsevier, reprinted from [83] with permission). (**c**) Influence of sidewall PCD and top PCD on thermal performance of GaN HEMTs with different GaN buffer thicknesses (from Gohel, K., 2024, doi:10.1088/1361-6641/ad4a66, CC-BY 4.0, reprinted from [84] with permission). (**d**) Device structure and breakdown characteristics of GaN HEMTs with a p-type diamond layer (Copyright 2016, IEEE, reprinted from [86] with permission). (**e**) Comparison of thermal characteristics among GaN HEMTs with different nanopatterns (from Wang, P., 2025, doi:10.3390/nano15080574, CC-BY 4.0, reprinted from [87] with permission). (**f**) f_T_ and f_max_ of diamond-capped GaN HEMTs with different h_d_ and ε_rd_ (Copyright 2022, IEEE, reprinted from [88] with permission).

Studies on AlGaN/GaN HEMTs have shown that a diamond heat spreader can significantly reduce the device junction temperature [75,76]. The main substrate materials used for GaN HEMTs include sapphire, Si, SiC, and diamond [77,78,79,80]. Zhang et al. performed three-dimensional thermal simulations of multi-finger AlGaN/GaN HEMTs using COMSOL 5.4a, finding that diamond thickness is positively correlated with temperature uniformity [81]. The heat dissipation effect of the top diamond varies a lot with the substrate type. Low TC substrates inherently have poor heat dissipation capabilities, and the additional vertical heat dissipation and lateral paths provided by the top diamond can significantly reduce the junction temperature, resulting in a more pronounced heat dissipation effect. Hang Zhang et al. simulated the diamond-capped GaN HEMTs and demonstrated that this structure can effectively reduce the dynamic steady-state peak junction temperature (T_ep_) by 8.8% to 33.9% with 1–14 μm thick diamond films [82]. As shown in Figure 7a, although thicker diamond layers yield better heat dissipation, this improvement trend gradually slows down. In 2024, Mohd Faizol Abdullah et al. from the *Universiti Kebangsaan Malaysia* conducted chip-packaging co-simulations, and they used Silvaco Victory Device and ANSYS Electronics Desktop Icepak software for this work [83]. Further research confirmed a key finding that replacing the top SiN_x_ passivation layer with a high-TC PCD of 500 W/(m·K) achieves significant thermal improvements, and this replacement is illustrated in Figure 7b. At a power density of 6.0 W/mm, the maximum junction temperature (T_j,max_) decreases from 105.8 °C to 98.2 °C, and, meanwhile, temperature uniformity (ΔT_j_) improves from 18% to 8%. This demonstrates that diamond can lower the junction temperature and enhance temperature distribution uniformity.

The heat transfer efficiency within the device is influenced by multiple factors, including the position of the diamond, the TBR of diamond/GaN, and the distance between the hot spot and the diamond. To explore the effects of these factors on the device junction temperature, Khush Gohel et al. from the University of Wisconsin-Madison performed multi-path extraction thermal simulations of diamond-capped AlGaN/GaN HEMTs using Silvaco [84]. Combined with the theoretical analysis of thermal stress between diamond and III-nitrides by Jerome A. Cuenca et al., the simulation parameters were extracted [85]. As shown in Figure 7c, a comparison between devices with and without a PCD-capped structure reveals significant differences in the heat dissipation effect of the sidewall PCD. When the top PCD is present, the sidewall PCD connects the top PCD heat dissipation layer and the single crystal diamond (SCD) substrate, thereby forming a new heat dissipation pathway and enhancing the thermal dissipation. The study also analyzed the effects of TBR at the top PCD/GaN interface, sidewall PCD/GaN interface, and SCD substrate/GaN interface. When the TBR of SCD substrate/GaN is a relatively low value of 3.1 m^2^·K/GW, an appropriate thinning of the GaN buffer layer can reduce the overall thermal resistance of the device. This is because the reduction in the thickness of the GaN buffer layer brings the GaN/SCD interface closer to the hot spot region [28]. However, this trend is weakened for devices with top PCD and sidewall PCD due to the newly added heat dissipation path.

In addition to the conventional diamond-capped GaN HEMTs, Tomás Palacios et al. from the Massachusetts Institute of Technology proposed a p-type diamond between the drain and gate of GaN HEMTs to optimize the device breakdown voltage (BV) while serving as a heat dissipation layer, with the structure shown in Figure 7d [86]. The p-type diamond has a thickness of 60 nm and a doping concentration of 1 × 10^18^ cm^−3^. Simulations indicate that this structure increases the BV by 3.5 times. This is due to the fact that the p-diamond capping layer can compensate the 2DEG in the off-state, enabling a more uniform electric field distribution and a lower electric field peak. In 2025, Qing Wang et al. from Southern University of Science and Technology conducted performance simulations on diamond-capped GaN HEMTs with nanopatterning [87]. They defined this grooved nanopatterned structure as a Trench Double-layer Passivation (TDP) structure. As shown in Figure 7e, compared with the single-passivated (SP) structure and double-passivated (DP) structure, the TDP structure achieves a temperature reduction of 43.01 °C and 18.22 °C, respectively. Meanwhile, its I_dss_ reaches 1.266 A/mm, which is 29.8% and 19.1% higher than that of the SP structure and DP structure. The maximum transconductance (g_m,max_) is approximately 4.78% and 1.86% higher than that of the SP structure and DP structure, reaching 0.329 S/mm. The on-resistance (R_on_) is 9.6% lower than that of the SP structure and 9.0% lower than that of the DP structure, with a minimum value of only 2.64 Ω·mm.

In addition to exerting a positive impact on the thermal and DC electrical properties of devices, the diamond-capped structure also influences their RF performance. In 2021, D. Nirmal et al. conducted a study on two-finger GaN HEMTs using TCAD software [89]. Owing to the suppression of the self-heating effects and the reduction in electron scattering, the peak g_m_ of the diamond-capped GaN HEMTs is 150 mS, which is approximately 14% higher than that of the conventional structure. In addition, the f_T_ is increased from 21.14 GHz to 25.36 GHz. In 2022, Xinyu Zhou et al. from Stanford University developed a co-simulation scheme [88]. Based on published experimental data of millimeter-wave N-polar GaN metal–insulator–semiconductor high-electron-mobility transistors (MIS-HEMTs), they investigated the impact of diamond as a passivation layer on the high-frequency characteristics of GaN HEMTs by combining the PathWave Advanced Design System (ADS) and Ansys High Frequency Structure Simulator (HFSS). The f_T_ and f_max_ of GaN HEMTs without any passivation are 106 GHz and 270 GHz, respectively. For traditional passivation schemes, SiN_x_ with a thickness of 50 nm and a relative dielectric constant of 7 reduces f_T_ to 86 GHz and f_max_ to 225 GHz. Benzocyclobutene (BCB), with a thickness of 2.7 μm and a relative dielectric constant of 2.65, exhibits more significant attenuation, with f_T_ = 84 GHz and f_max_ = 217 GHz. In contrast, for GaN HEMTs with diamond as the passivation layer, as shown in Figure 7f, when the thickness of the diamond passivation layer (h_d_) ranges from 0.6 μm to 2.7 μm, f_T_ decreases from 98 GHz to 85 GHz. The key reason is that the diamond passivation acts as a parallel-plate capacitor. The capacitance is expressed as $C=\frac{\varepsilon_{rd}S}{h_{d}}$, where *S* denotes the surface area of the device and *ε_rd_* is the relative dielectric constant of diamond. When *ε_rd_* and *S* are constant, *C* is inversely proportional to *h_d_*; increasing *h_d_* raises the passivation capacitance. According to the small signal model formula, it directly reduces f_T_. When εrd increases from 2.3 to 4.0 with an *h_d_* of 1.0 μm, f_T_ drops by 5.2% from 96 GHz to 91 GHz, and f_max_ drops by 6.6% from 243 GHz to 227 GHz. In conclusion, diamond passivation balances the controllable high-frequency attenuation and superior thermal conductivity.

## 4. Key Fabrication Processes

Integrating diamond-capped GaN HEMTs presents several critical challenges, foremost among which is the compatibility between high-temperature diamond growth and GaN HEMT fabrication processes, in addition to diamond etching. This contradiction arises primarily from incompatible process conditions, with diamond CVD typically requiring a high-temperature environment of 600–800 °C that conflicts with the operational stability of GaN HEMTs. Specifically, conventional Schottky gate metals such as Ni/Au lack a sufficient thermal tolerance at such temperatures. This process incompatibility directly gives rise to a cascade of critical performance issues in GaN HEMTs. Elevated temperatures damage the gate electrode and reduce its gate control capability, leading to an increased gate leakage current [90,91]. Simultaneously, the harsh growth environment degrades carrier transport in the GaN channel, resulting in substantial reductions in the output current and switching speed that compromise the intrinsic high-frequency and high-power capabilities of GaN HEMTs essential for their RF and power applications [92]. In recent years, numerous studies have provided experimental support for the feasibility of this integration. The key process solutions are as follows. One is the gate-last process, which is growing diamond before gate fabrication. The other is the gate-first process, which is depositing diamond after gate fabrication, and the gate-first process usually requires high-temperature-resistant gate metal materials or an MIS structure. Additionally, the diamond etching process, including multistep etching, cyclic etching and combined dry/wet etching schemes, is also significant for device performance enhancement.

### 4.1. Diamond Etching

Diamond etching, as one of the key processes for the integration of diamond-capped GaN HEMTs, is the focused research topic. The quality of diamond etching directly influences electrode contact formation, device electrical performance, and final heat dissipation efficiency. As early as 2004, Hwang et al. conducted research on diamond etching using inductively coupled plasma (ICP) etching [93].

**Figure 8:**
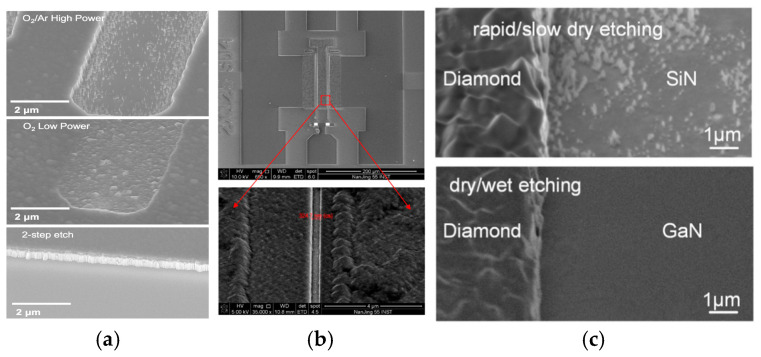
(**a**) SEM images of gate fingers after different etching treatments (Copyright 2016, IOP Publishing, reprinted from [94] with permission). (**b**) Etching quality of the NCD capping layers for gate region (from Guo, H., 2022, doi:10.3390/mi13091486, CC-BY 4.0, reprinted from [96] with permission). (**c**) SEM images of the samples with rapid/slow dry etching and dry/wet combined etching process (Copyright 2023, Elsevier, reprinted from [97] with permission).

In 2016, the U.S. Naval Research Laboratory proposed a two-step etching method to address the challenges of diamond etching [94]. They adopted SiN_x_ as the hard mask. First, they removed approximately 90% of the diamond with high-power O_2_/Ar ICP etching and then eliminated the remaining 10% through low-power O_2_ ICP etching. As shown in Figure 8a, for the single high-power O_2_/Ar ICP etching, it brings micro-mask effects. In contrast, low-power O_2_ ICP etching has an etch rate of only one-tenth that of high-power etching and can fully preserve the original grain structure of NCD without obvious micro-mask effects. Combining these two stages, the two-step etching can not only achieve vertical sidewalls but also a flat bottom surface, perfectly balancing the etch efficiency and surface quality. In 2018, Michel Challier et al. from Saarland University in Germany proposed a cyclic etching method that alternately uses different gas combinations to etch diamond [95]. They adopted quartz masks with optimized 30° sidewalls and designed two alternating plasma sequences with cooling steps inserted during the etching. Using Ar, O_2_ and SF_6_ as the etching gases, the method achieved a smooth diamond surface with an RMS less than 1 nm. In 2022, Guo et al. from the Nanjing Institute of Electronic Devices innovatively achieved diamond etching with a precision of 0.5 μm and successfully fabricated AlGaN/GaN HEMTs with a gate length of 0.5 μm [96]. The core of this process lies in the adoption of multi-step etching technology for the gate region. First, O_2_/Ar is used for the rapid etching to ensure the verticality of the trench. Second, high-quality surface etching is performed using ICP with O_2_ to mitigate burrs on the etched surface. Finally, low-power etching of the isolation layer is conducted with O_2_. As shown in Figure 8b, the multi-step etching technology successfully achieves a high-quality gate region with a flat etched surface and no obvious defects, which intuitively verifies the reliability of the process. The I_dss_ of the diamond-capped AlGaN/GaN HEMTs fabricated using their method is 950.45 mA/mm at V_GS_ = 1 V, which is 27.9% higher in output characteristics compared to traditional AlGaN/GaN HEMTs. The f_T_ reached 34.6 GHz, with an increase of 1.8%, proving that the diamond thin film does not introduce significant adverse effects such as parasitic capacitance. At a frequency of 10 GHz, the average gain of the diamond-capped AlGaN/GaN HEMTs is 36.7% higher than that of traditional AlGaN/GaN HEMTs, which confirms that the diamond-capped AlGaN/GaN HEMTs have an excellent RF performance.

Moreover, the diamond etching process often faces an incomplete etching of the SiN_x_ hard mask, and residual micro-masks can hinder the complete etching of diamond. The removal of the SiN_x_ interlayer through F-based ICP can also cause etching damage to the AlGaN surface. To address the above issues, Mei Wu et al. proposed a scheme combining the dry etching of diamond with the wet etching of SiN_x_ [97]. First, the diamond is subjected to sequential fast and slow dry etching steps to ensure the diamond etching rate while precisely stopping at the SiN_x_ interlayer, avoiding damage to the underlying structure. Subsequently, wet etching is performed using a diluted HF solution to remove the residual SiN_x_ hard mask from the previous step, and, at the same time, the SiN_x_ interlayer with an initial thickness of 20 nm serving as the etch stop layer is stripped in the device active regions. Eventually, as shown in Figure 8c, compared with the conventional rapid/slow dry etching process, the dry/wet combined etching process adopted by the researchers achieved a smoother, damage-free AlGaN/GaN surface without residual pillars. The off-state drain current of the diamond-capped device is 3.49 × 10^−4^ mA/mm, nearly identical to that of the reference device. Meanwhile, the peak g_m_ is significantly enhanced from 117.2 mS/mm to 127.6 mS/mm. This is attributed to the smooth and damage-free AlGaN/GaN surface, which reduces carrier scattering and facilitates efficient electron transport. The self-heating effects show a reduction of approximately 42.6%, indicating a weakened self-heating effect.

### 4.2. Process Integration Routes of Diamond-Capped GaN HEMTs

Based on the key etching process, researchers have developed different process integration routes to balance heat dissipation and device performance. The integration approaches of diamond-capped GaN HEMTs are mainly categorized into two types, which are gate-first and gate-last. The gate-last approach involves depositing and etching diamond first, followed by subsequent GaN device fabrication processes, which can avoid the direct impact of high temperatures during diamond growth on the gate. The advantage of the gate-first approach lies in its ability to directly fabricate the entire device on GaN. However, it faces the challenge of gate damage caused by high temperatures. In the following sections, we will separately introduce the diamond-capped devices fabricated via these two routes.

**Figure 9:**
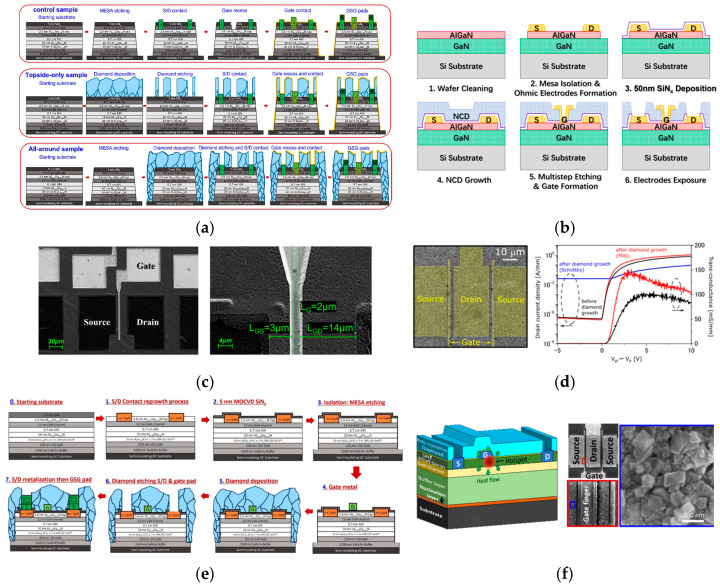
(**a**) Fabrication processes of the control, topside-only and all-around diamond-integrated samples (Copyright 2021, IEEE, reprinted from [99] with permission). The NCD-capped GaN HEMT (from Fu, Y., 2025, doi:10.3390/cryst15030242, CC-BY 4.0, reprinted from [100] with permission): (**b**) schematic diagram of its fabrication processes, (**c**) SEM image labeled with its parameters. (**d**) SEM image of GaN MIS-HEMTs with capped diamond film and the comparison of transfer curves (Copyright 2021, IOP Publishing, reprinted from [101] with permission). (**e**) Fabrication step sequence of all-around diamond-capped N-polar GaN MIS-HEMTs with Mo gate (from Soman, R., 2025, doi:10.35848/1882-0786/adcb87, CC-BY 4.0, reprinted from [102] with permission). (**f**) Schematic diagram and SEM image of diamond-capped AlGaN/GaN HEMTs on SiC (Copyright 2025, AIP Publishing, reprinted from [103] with permission).

As early as 2012, T.J. Anderson et al. proposed the gate-last approach and characterized the performance of NCD-capped AlGaN/GaN HEMTs [98]. The I_dss_ of the GaN HEMTs fabricated through this process reached 249.6 mA/mm at V_DS_ = 20 V, which is higher than the 206.1 mA/mm of the control sample. At a DC power of 1 W, the calculated gate peak temperatures of GaN HEMTs with 0.5 μm and 2 μm NCD capping layers are approximately 20.9% and 38.9% lower, respectively, compared to the control sample. In 2022, Srabanti Chowdhury et al. from Stanford University proposed a fabrication scheme for all-around diamond GaN HEMTs [99]. As shown in Figure 9a, the process flow begins with mesa etching down to the SiC substrate, followed by diamond growth on the sidewalls and top surface. The diamond grown on the sidewalls connects the top diamond layer with the SiC substrate, forming an efficient heat dissipation pathway from the channel to the substrate, thus resulting in an excellent heat dissipation performance. The heat dissipation effect of the 500 nm thick all-around diamond structure is comparable to that of the 2.2 μm thick topside-only diamond structure, demonstrating the superior characteristics of the fully enclosed structure. Meanwhile, the device temperature was characterized through gate resistance measurements. As shown in Figure 9b, when operating at a DC power density of 9.5 W/mm, the gate electrode temperature of the fully enclosed structure device was reduced by 98 ± 19 °C compared to GaN HEMTs without the diamond structure. In 2025, Fu Yu et al. from Xidian University fabricated T-gate GaN HEMTs with a nanodiamond-capped passivation layer grown at 650 °C [100]. The device fabrication process and SEM of the device are shown in Figure 9c and Figure 9d, respectively. The I_dss_ of the device is 24% higher than that of the traditional device, and R_on_ decreased from 19.9 Ω·mm to 13.2 Ω·mm. Benefiting from the optimized electric field distribution enabled by the T-gate structure with a diamond passivation layer, the breakdown voltage (BV) was increased from 400 V to 500 V. Meanwhile, the junction temperature–power density slope of the unpassivated device is 25.88 °C/(W·mm), while that of the NCD-passivated device is only 16.38 °C/(W·mm), representing a 36% improvement in heat dissipation efficiency.

However, for the gate-first process route, the core challenge is that both the gate metal and the source and drain ohmic metal must withstand the high temperatures required for subsequent diamond growth. In 2016, T.J. Anderson et al. proposed the sacrificial gate process, introducing a 10 nm thick Al_2_O_3_ layer that serves both as a protective layer and an etch stop layer for SiN_x_ [104]. This process is suitable for scenarios where the diamond film exhibits a non-uniform thickness. In recent years, significant progress has been achieved in realizing high-performance short-gate-length devices. In 2021, Junya Yaita et al. from Fujitsu Limited fabricated microcrystalline diamond (MCD)-capped GaN MIS-HEMTs [101]. The MIS gate ensures the device’s compatibility with the 700 °C high-temperature growth process of the MCD film, avoiding the degradation of traditional Schottky gate structures at high temperatures [105]. As shown in Figure 9d, the device exhibits an on/off ratio of 10^6^, while the I_dss_ increased from 0.9 A/mm to 1.1 A/mm and the g_m_ improved from 102 mS/mm to 148 mS/mm. At a DC power density of 25 W/mm, the maximum temperature of the device is reduced by 100 K. In 2025, Srabanti Chowdhury et al. from Stanford University fabricated fully enclosed diamond N-polar GaN MIS-HEMTs with a gate length of only 150 nm [102]. The fabrication process is shown in Figure 9e. Molybdenum (Mo) was used as the gate metal, and, when combined with the MIS structure, it is compatible with low-temperature diamond growth processes, which can reduce the thermal damage to the gate and the 2DEG. The device exhibits an I_dss_ of 0.96 A/mm and an on/off ratio of 10^5^. The study verified the feasibility of integrating the MIS gate structure with diamond, providing key technical support for the thermal management of GaN HEMTs. In 2025, the same group investigated the characteristics of AlGaN/GaN HEMTs with a two-finger T-gate structure, as shown in Figure 9f [103]. Compared with devices without diamond integration, f_T_ remained unchanged of 23 GHz after integration, while f_max_ decreased by 8.3%, primarily attributed to an increase in gate resistance, which rises from 15 Ω to 25 Ω. The g_m_ increased by 30 mS/mm from 320 to 350 mS/mm, the I_dss_ rises by 15.5%, and the current collapse increases only slightly from 12.1% to 14.5%. Under the optimized diamond growth temperature in the range of 450 to 500 °C, the gate leakage current increased by only 4.8 times compared with devices without diamond integration. However, it can be significantly reduced through annealing. At a DC power density of 24 W/mm, the channel temperature of the diamond-capped GaN HEMTs is decreased by 111 °C compared with GaN HEMTs without diamond.

## 5. Future Perspectives

Continued advances in diamond-capped GaN HEMT thermal management are expected to fundamentally address the self-heating issues in high-frequency and high-power GaN HMETs, thereby improving their stability and overall performance. Future research should focus on key technical challenges, particularly in the areas of diamond growth and interface engineering. Optimized plasma parameters and adjusted carbon and hydrogen dilution ratios may enable the co-optimization of low growth temperature and high thermal conductivity. Building on recent progress in reducing the TBR, the use of ultrathin interlayers and nanoscale patterning could further improve interface compatibility, lower TBR, and enhance interfacial bonding strength, ensuring structural and thermal stability under prolonged high-temperature and high-power operation. In addition, the smart cut technique could be explored to directly transfer high thermal conductivity single-crystal diamond onto the device surface as a top diamond capping layer, further improving heat dissipation capabilities [106,107].

In terms of device simulation, future efforts should focus on developing multiphysics models that simultaneously account for the temperature field, electric field, and stress field to better understand the effects of thermal stress on electric field distribution and carrier transport. Dynamic thermal analysis methods suitable for high-frequency switching conditions should also be developed, and long-term reliability models incorporating material degradation mechanisms should be established to provide more targeted guidance for device structure optimization and interface design.

In device fabrication, process development should target the unique properties of diamond to achieve high-precision and low-damage patterning with a good selectivity and sidewall quality. For integration, improvements in gate-last process efficiency, combined with the development of thermally robust gate stacks and ultra-low-temperature diamond growth, will enhance the compatibility with the gate-first process.

## 6. Conclusions

This paper systematically summarizes the latest progress in the heat dissipation technology of diamond-capped GaN HEMTs. It centers on three core aspects, including the diamond growth and TBR optimization, device simulation, and key manufacturing processes. It comprehensively summarizes the latest advancements, from material growth to actual device fabrication, providing a complete technical framework for solving the self-heating problem of GaN HEMTs with diamond-capped heat spreaders.

In terms of diamond growth and interface optimization, nucleation technology primarily relies on the electrostatic seeding method, supplemented by micro–nano-seed mixed nucleation technology. The former can achieve a high nucleation density of 10^13^ cm^−2^, while the latter further reduces TBR by filling interfacial voids. Meanwhile, for the growth stage optimization, multi-stage MPCVD parameter regulation and growth equipment optimization are adopted for the achievement of the thin diamond film with high TC. By introducing O_2_ in the growth process, growth temperature can be reduced to 400 °C, which resolves the contradiction between high temperature and device compatibility. For the TBR optimization, research mainly focuses on reducing the interlayer material thickness, investigating carbon diffusion mechanisms, and designing arrayed structures. And, finally, the TBR of the diamond/GaN interface can be as low as 3.1 ± 0.7 m^2^·K/GW. In addition, device simulations provide a theoretical analysis for the heat dissipation of diamond-capped GaN HEMTs with various parameters and find the modulating trends for the guidance of the device fabrication. For the key fabrication process of diamond-capped GaN HEMTs, diamond etching is widely studied, and the two-step etching or dry/wet etching method can solve the problems of surface damage and residual pillars in traditional etching. Meanwhile, for the device integration process, the routes can be divided into two types, which are called gate-first and gate-last. The gate-last process is more commonly adopted, as it eliminates the need to consider the compatibility between the gate metal and the high-temperature growth of diamond. However, the gate-first process is more conducive to the large-area deposition of diamond, which can effectively improve the heat dissipation efficiency. This can be achieved by adopting gate metal with a high temperature resistance, introducing MIS structures, or reducing the growth temperature of diamond. Future research should continuously focus on the optimization of the diamond TC with lower temperature, the TBR, the thermal structure design, and the device fabrication process. It is believed that, with continueous advances in these fields, the diamond capping technology for GaN HEMTs will exhibit tremendous prospects in high-frequency and high-power applications.

## Data Availability

No new data were created or analyzed in this study. Data sharing is not applicable to this article.
